# Cysteine string protein (CSP) and its role in preventing neurodegeneration

**DOI:** 10.1016/j.semcdb.2015.03.008

**Published:** 2015-04

**Authors:** Robert D. Burgoyne, Alan Morgan

**Affiliations:** Department of Cellular and Molecular Physiology, Institute of Translational Medicine, University of Liverpool, Crown St., Liverpool L69 3BX, UK

**Keywords:** ANCL, adult onset neuronal ceroid lipofuscinosis, CREB, cAMP response element binding protein, CSP, cysteine string protein, GABA, gamma-amino butyric acid, SGT, small glutamine-rich tetratricopeptide repeat-containing protein, SNAP-25, synaptosome-associated protein of 25-kDa, SNAREs, soluble NSF attachment protein receptors, VAMP, vesicle associated membrane protein, *DNAJC5*, SNAREs, SNAP-25, Adult onset neuronal ceroid lipofuscinosis, Alzheimer's disease, Resveratrol

## Abstract

Cysteine string protein (CSP) is a member of the DnaJ/Hsp40 family of co-chaperones that localises to neuronal synaptic vesicles. Its name derives from the possession of a string of 12–15 cysteine residues, palmitoylation of which is required for targeting to post-Golgi membranes. The DnaJ domain of CSP enables it to bind client proteins and recruit Hsc70 chaperones, thereby contributing to the maintenance of protein folding in the presynaptic compartment. Mutation of CSP in flies, worms and mice reduces lifespan and causes synaptic dysfunction and neurodegeneration. Furthermore, recent studies have revealed that the neurodegenerative disease, adult onset neuronal ceroid lipofuscinosis, is caused by mutations in the human CSPα-encoding *DNAJC5* gene. Accumulating evidence suggests that the major mechanism by which CSP prevents neurodegeneration is by maintaining the conformation of SNAP-25, thereby facilitating its entry into the membrane-fusing SNARE complex. In this review, we focus on the role of CSP in preventing neurodegeneration and discuss how recent studies of this universal neuroprotective chaperone are being translated into potential novel therapeutics for neurodegenerative diseases.

## 1 Introduction: discovery and properties of CSPs

Cysteine string protein (CSP) was first discovered in *Drosophila melanogaster* based on the use of a neuronal-specific monoclonal antibody that labelled nerve terminals. Cloning of cDNA revealed that the antibody recognised proteins encoded by three splice variants of a novel gene. These were distinguished by the presence of a string of 11 contiguous cysteine residues leading to the naming of the protein(s) as cysteine string protein [1]. Soon after, a CSP was also discovered in *Torpedo californica* following an attempt to identify possible subunits of neuronal calcium channels, which at that time had not been identified or molecularly characterised [2], and it was shown to be localised to synaptic vesicles [3]. CSPs were soon identified in mammalian species [4–6]. The *Torpedo* protein [7] and subsequently CSPs in other species were found to be extensively palmitoylated on their cysteine residues. Palmitoylation was shown to determine the membrane association of CSPs [8–10] and the enzymatic basis for palmitoylation of CSP has been extensively studied [11–16]. In addition to being expressed in multiple species, CSP was also found to be present on a wide range of types of secretory vesicles in neuronal and non-neuronal tissues [5,6,17–19]. Invertebrates such as *Drosophila* and *Caenorhabditis elegans* have only a single CSP-encoding gene (*Csp* and *dnj-14*, respectively); whereas mammals express three CSP proteins (α, β, γ) that are encoded by the *DNAJC5a*, *b* and *g* genes. CSPα and CSPβ are highly homologous throughout their amino acid sequences, but CSPγ is more distantly related [20]. CSPα is the major protein expressed in most cells and virtually all neurons. In contrast, mammalian CSPβ and CSPγ are expressed only in testis [21,22], with the exception that CSPβ is uniquely also expressed in auditory hair cell neurons [23,24]. The functional roles of CSPβ and CSPγ are unknown.

**Fig. 1:**
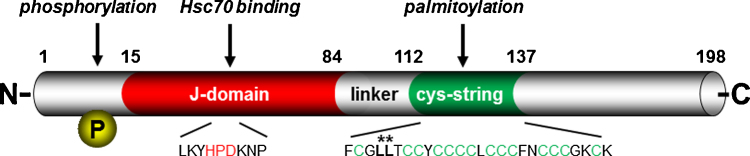
Domain structure of human CSPα. The locations of the Serine10 phosphorylation site, the HPD motif in the J domain, and the cysteine-rich region of the protein are indicated. The leucine residues mutated in ANCL patients are highlighted by asterisks.

The sequence of CSP revealed that it contains a conserved J domain (Fig. 1) and so it is a member of the DnaJ/Hsp40 family of co-chaperones. CSP has a characteristic HPD motif in the J-domain that is required in this family of proteins to allow them to bind to Hsc70/Hsp70 and act in concert with Hsc70/Hsp70 in the refolding or disaggregation of client proteins. Biochemical analysis of CSP demonstrated that it could indeed bind to and activate the ATPase activity of Hsc70 [25,26] and also that it could reverse aggregation of model substrates in conjunction with Hsc70 [27]. It was subsequently suggested that CSP and Hsc70 function along with small glutamine-rich tetratricopeptide repeat-containing protein (SGT) in a trimeric complex [28,29]. While CSP is clearly able to bind to SGT, there are doubts about the physiological significance of the SGT interaction. These stem from the fact that the interaction of SGT with CSP characterised in vitro with recombinant proteins and via the yeast 2-hybrid method occurs through the non-palmitoylated cysteine-string domain [29], which would not be available for interaction in vivo. Recently it has been established that SGT is a chaperone for the transmembrane region of tail-anchored proteins [30,31], suggesting that SGT binding to recombinant CSP in vitro may be a consequence of its artefactual recognition of the hydrophobic cysteine rich region due to its similarity to a bona fide transmembrane domain.

## 2 Which proteins are substrates for refolding by the synaptic chaperone CSP?

Early efforts to discover potential clients for CSP identified multiple direct protein interactions including with syntaxin [32–34], VAMP [35,36], G protein subunits [37,38], Rab-GDI [39], mutant huntingtin [40,41], the α1B subunit of N-type calcium channels [37,42], the α1A subunit of P/Q type calcium channels [43] and synaptotagmins [44,45]. The observed reduction of SNAP-25 in CSPα knock out mice led to the identification of this protein as a potential CSPα client [46]. More recent work has attempted to identify novel CSPα clients by using an unbiased proteomic approach searching for any proteins down-regulated in the brains of CSPα knock-out mice. This resulted in the identification of 10 chaperones and 27 additional down-regulated proteins that included SNAP-25 [47]. The identification of one novel CSPα client, dynamin 1, was confirmed in direct binding assays and a role for CSPα in dynamin-mediated synaptic vesicle recycling was described in another study [48].

## 3 Cellular functions of CSP

Information on the functional importance of CSP came initially from analysis of *Csp* mutants in *Drosophila*. The first of the papers on these mutants provided key evidence supporting the now accepted role of CSP as a neuroprotective chaperone at the synapse [49–51]. In this work it was found that loss of CSP expression resulted in very rapid death of adult flies, by 5 days at 22 °C and within 1 h at 29 °C, and the flies showed evidence of synaptic degeneration observed by electron microscopy [52]. The follow-up analyses of these mutant flies focused to a large extent on studying the phenotype of the few adult survivors (95% of *Csp* null mutants die during development) and in particular there was an emphasis on what was seen as the “temperature-dependent” aspects of the phenotypes, where the flies rapidly became paralysed at elevated temperature [53], rather than the causes of the rapid neurodegeneration and death.

Electrophysiological analysis of synaptic function in *Csp* mutant flies suggested that CSP was required for normal synchronous evoked neurotransmitter release [54,55], but this did not appear to be related to any effect on presynaptic calcium channel function [56]. In addition, the flies showed various defects in calcium handling or calcium coupling to exocytosis [57–60]. Studies on non-neuronal cells, examining the effects of CSP overexpression suggested a direct role for CSP in regulated exocytosis [61–64]. The ability of CSP to interact with its client proteins syntaxin or synaptotagmin in vitro was found to be modulated by its phosphorylation on Serine10 [44,65] and this phosphorylation allows interaction of CSP with 14-3-3 protein [66]. Ser10 phosphorylation on CSP appears to be constitutive within neuronal tissues [67–69], although the extent of phosphorylation varies greatly between neighbouring GABAergic and glutamatergic synapses within the same region of the cerebellum [67]. The effects of CSP over-expression on the late stages of regulated exocytosis are modulated by its phosphorylation on Ser10 [65,70,71].

## 4 CSP and neurodegeneration

The key neuroprotective role of CSP was apparent in the fly mutants but only rose to prominence in considerations of the function of the protein following analysis of CSPα knock-out mice. For the first few weeks of life, these mice appeared normal and did not show defects in neurotransmission [22], but then developed a progressive sensorimotor disorder, evidence of neurodegeneration in neuromuscular junctions and calyx synapses and died at around 8 weeks of age. These mice also showed extensive and rapid degeneration of retinal photoreceptors [23] and hippocampal GABAergic neurons were found to be differentially sensitive to the absence of CSPα compared to glutamatergic neurons [72]. The high sensitivity of GABAergic neurons was attributed to their high levels of synaptic activity, which is consistent with the more rapid degeneration seen in tonically active ribbon synapses of photoreceptor cells compared to other retinal synapses [23]. More detailed analysis of the mice revealed additional defects in synaptic function including a reduction in the normal calcium sensitivity of the neurotransmitter release mechanism [73] and also an impairment of synaptic vesicle recycling at the neuromuscular junction [48]. These latter findings would fit with the existence of multiple client proteins for CSP including dynamin 1.

The evolutionary conservation of CSP's neuroprotective function is underlined by a recent study of the *C. elegans* CSP orthologue, which is known as DNJ-14. The phenotypes of *dnj-14* null mutant worms show remarkable similarities to CSP knockout mice in that young animals are virtually indistinguishable from wild-type, but exhibit progressive neuronal dysfunction and neurodegeneration with increasing age and have a reduced lifespan [24]. It is not clear why complete loss of CSP in *Drosophila* causes ∼95% embryonic lethality, whereas no major effects on fertility or initial viability are observed in knockout worms and mice, but perhaps this reflects some specific functions of CSP during fly development. Neurodegeneration in aged *C. elegans dnj-14* mutants preferentially affects sensory neurons and indeed these animals exhibit severely impaired chemosensory neuron function before neurodegeneration becomes apparent [24]. This progression from early functional synaptic defects leading on to later structural alterations and neuronal death is reminiscent of many neurodegenerative diseases and suggests that the *dnj-14* worm model may be useful for identifying generally neuroprotective mechanisms and therapies. However, the surprising observation that loss of CSP is actually neuroprotective in a *Drosophila* model of injury-induced axonal degeneration [74] suggests that some acute neurodegeneration models could involve distinct mechanisms that may not be ameliorated by CSP's otherwise universal neuroprotective function.

A surprising finding was that the neurodegeneration and reduced life-span in CSPα KO mice was significantly reversed by overexpressing α-synuclein [46]. Both wild-type and a disease-related mutant (A30P) α-synuclein had positive effects although that of the wild-type protein was more marked. Conversely, the CSPα KO phenotype was exacerbated by knock-out of α- or β-synuclein [46]. Analysis of CSPα KO mice expressing the A30P mutant version of α-synuclein revealed that these mice show increased synaptic function consistent with a protective function even of this pathogenic form of α-synuclein [75]. A further important observation from the study of CSPα KO mice and α-synuclein overexpression was that the absence of CSPα resulted in a reduction in SNAP-25 levels and a corresponding reduction in the levels of assembled SNARE complexes [46]. The loss of SNAP-25 was not affected by overexpression of α-synuclein. In contrast, wild type but not A30P α-synuclein reversed the reduction in SNARE complexes suggesting that this may contribute, at least in part, to the protective effect of α-synuclein. A further link between α-synuclein and CSPα was shown in a study examining the effects of exogenous α-synuclein or a β-amyloid peptide on cultured hippocampal neurons where both treatments were observed to result in a reduction in levels of CSPα expression [76].

**Fig. 2:**
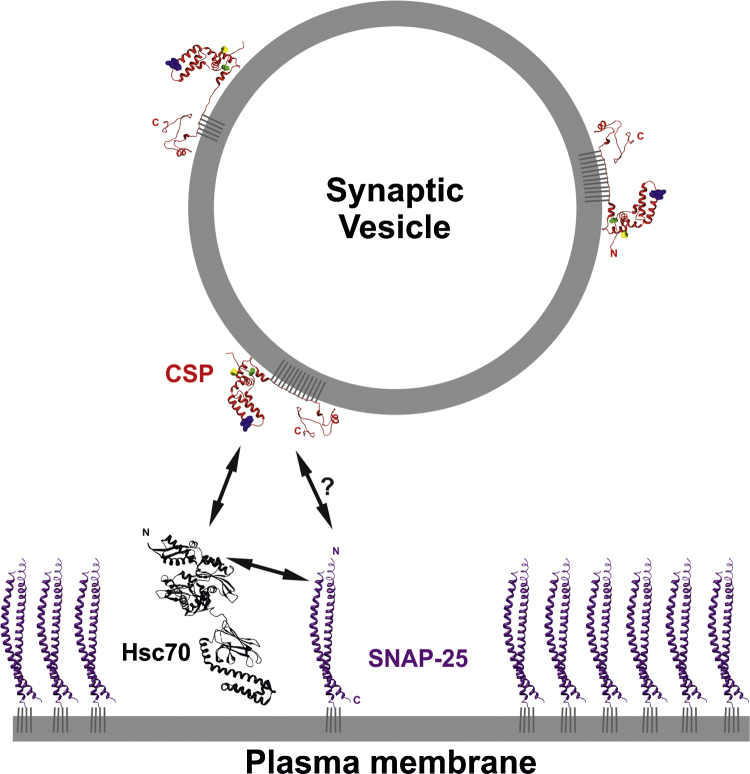
Chaperoning of SNAP-25 by CSP and Hsc70 at the synapse. A typical synaptic vesicle containing approximately three molecules of CSP [77] is represented. Binding of Hsc70 to SNAP-25 is thought to recruit CSP, thereby altering the conformation of SNAP-25, facilitating its entry onto the SNARE complex that drives membrane fusion. Direct binding is indicated by double-headed arrows. Structures of SNAP-25 (3IPD), and hsc70/DnaK (2KHO) were obtained from the Brookhaven protein data bank. The CSP structure prediction was generated by I-TASSER. Residues involved in Hsc70 binding (HPD motif, blue), synaptotagmin binding (E93, yellow), and the serine-10 phosphorylation site (green) are highlighted as spheres. All structures were rendered using UCSF chimaera.

It may seem surprising that SNAP-25 has been identified as a potential client for CSPα, as CSPα is primarily a synaptic vesicle protein whereas SNAP-25 is largely localised to the axonal plasma membrane (Fig. 2). Furthermore, CSPα is expressed at relatively low levels (2.8 molecules per synaptic vesicle and 941 molecules per synapse, on average) compared to the very abundant SNAP-25 (26,686 molecules per synapse) [77,78]. This raises some interesting mechanistic questions, as presumably only SNAP-25 molecules in the immediate proximity of tethered/docked vesicles at the active zone would be close enough to be chaperoned by CSPα/Hsc70. Perhaps this forms a useful checkpoint system, ensuring that the energy of ATP hydrolysis by Hsc70 is only expended on priming SNAP-25 molecules that can form a local SNARE complex and so drive membrane fusion. Nevertheless, it remains unclear how CSP prevents proteasomal degradation of the majority of the cellular pool of SNAP-25 despite its substrate being >25-fold in excess and mostly localised away from the active zone where CSP binding could potentially occur.

Notwithstanding these questions, detailed analysis of the CSPα knock-out mice has strongly suggested that SNAP-25 is the major client for CSP, that CSPα stimulates SNARE complex assembly and that neurodegeneration in the absence of CSPα can be explained by defective SNAP-25 function [79,80]. The work of Sharma et al. [79] showed that in the absence of CSPα SNAP-25 is selectively decreased due to its ubiquitination and its proteolysis in an activity-dependent manner. Moreover, CSPα over-expression increased SNAP-25 levels. In vitro binding experiments suggested that CSPα did not bind directly to SNAP-25, but did so indirectly via Hsc70. This is unusual as J domain proteins usually bind the client protein directly and then subsequently recruit Hsc70. In an additional study exploring synuclein function it was found that the absence of the synucleins reduced the ability of the SNAREs to assemble into SNARE complexes and that α-synuclein directly stimulated SNARE complex assembly in vitro. This required the direct binding of α-synuclein to VAMP. These findings could explain the earlier finding [46] that over-expression of α-synuclein compensates for the loss of CSPα and thereby SNAP-25. A key question at this point was whether the effect of CSPα absence on SNAP-25 levels or on its folding is the cause of neurodegeneration in the CSPα knock-out mice. One possibility was that it is not the reduction in SNAP-25 levels themselves that leads to synaptic dysfunction and subsequent neurodegeneration, but the accumulation of aberrantly folded forms of SNAP-25 that undergo abnormal protein interactions or that other CSPα clients were involved [81]. This issue of the link between SNAP-25 and neurodegeneration was further addressed leading to the finding that decreased SNAP-25 levels worsened the CSPα knock-out phenotype reducing life-span and levels of assembled SNARE complexes and exacerbated neurodegeneration [80]. An increase of SNAP-25 levels using lentiviral expression rescued the CSPα knock-out phenotype with neurodegeneration (neuronal loss) being reversed in brain regions over-expressing SNAP-25. These findings are consistent with SNAP-25 being the main CSPα client whose loss leads to neurodegeneration in the absence of CSPα.

## 5 CSP and human neurodegenerative disease

Despite the compelling evidence for an essential role of CSP in preventing neurodegeneration in animal models, a direct link to human disease was established only recently. Between 2011 and 2013, four independent research groups reported an association between mutations in the CSPα-encoding *DNAJC5* gene and adult onset neuronal ceroid lipofuscinosis (ANCL), using a combination of whole exome sequencing, linkage analysis and candidate gene resequencing [82–85]. ANCL, also known as autosomal dominant Kufs’ disease and Parry disease, is a very rare hereditary neurodegenerative disorder. It presents with broad clinical variability, although common signs include generalised epilepsy, movement disorders and progressive dementia. The disease has a mean age of onset of 30 years and progresses rapidly upon diagnosis, with death occurring on average at 45 years of age [85]. Pathologically, ANCL is associated with intra-neuronal inclusions of autofluorescent lipofuscin-like material and neurodegeneration, hence its classification as one of the neuronal ceroid lipofuscinoses, which is a large genetically heterogeneous class of neurodegenerative disorders defined by these two essential features [86,87]. However, the visual system tends to be unaffected by neurodegeneration in ANCL, in contrast to most other NCLs that are generally associated with blindness [85,86]. Given its rarity and clinical variability, ANCL is often misdiagnosed [83], so it is important that several independent groups identified the same *DNAJC5* mutations in different patient groups, leaving little doubt that these mutations cause ANCL.

Interestingly, all ANCL patients studied to date harbour one of two mutations in the coding sequence for the cysteine string motif, resulting in either a deletion of leucine116 or a leucine115-arginine substitution [82–85] (see Fig. 1). Given the known requirement of the cysteine string domain for palmitoylation [7,9,10] and for targeting to post-Golgi membranes [9], this immediately suggested that the mutant proteins may be inefficiently targeted to synaptic vesicles. Indeed, the original report by Noskova et al. [82] showed that recombinant GFP-tagged CSPα mutant constructs were retained in an abnormal cell body localisation co-migrating with endoplasmic reticulum and Golgi markers, in contrast to wild type GFP-CSPα that was efficiently transported to the plasma membrane. Consistent with such a targeting defect, post-mortem brain samples from ANCL patients similarly showed a large reduction in synaptic CSPα levels compared to controls [82]. These findings were independently confirmed and extended by Greaves et al. [88], who demonstrated that the ANCL mutations induce aggregation into SDS-resistant palmitoylated aggregates. Importantly, Greaves et al. used a dual epitope tagging system to reveal that wild type CSPα can co-aggregate with mutant CSPα [88], a finding that has recently been confirmed [89], thereby potentially explaining the dominant effect of the mutations and the otherwise puzzling observation that ANCL patients lack synaptic CSPα protein despite carrying one wild type *DNAJC5* allele.

Although ANCL is the only disease known to be caused by CSP mutations, alterations in the levels or activity of CSP could potentially impact on other neurodegenerative conditions given its neuroprotective function. One clue is the finding that CSPα can interact with mutant huntingtin containing an expanded polyQ domain but not the wild type protein in vitro [40]. This was confirmed in a proteomic study of mouse brain [41] but its relevance to the progress of Huntington's disease is still to be established. In addition, it has recently been reported that expression of CSPα is reduced in degenerating areas of the forebrain in post-mortem samples of Alzheimer's patients [90]. It appears that this is not simply a consequence of reduced synaptic vesicle number, as the integral synaptic membrane protein marker, synaptophysin, did not exhibit equivalent reduced expression. Interestingly, the reduction of CSPα expression is restricted to degenerating brain areas, with levels actually being increased in non-degenerating cerebellar tissue from the same patients [90]. Note, however, that heterozygous CSP KO mice, which contain half the normal CSP protein level, do not show any difference in susceptibility to prion disease in the ME7 model [91]. Nevertheless, a further connection between CSPα and more common forms of neurodegeneration comes from the observation that SNARE complex levels are reduced in post-mortem brain samples from both Alzheimer's and Parkinson's disease patients [92]. Given the role of CSPα in facilitating SNARE complex assembly discussed earlier, it is tempting to speculate that the reduced levels of SNARE complexes are a consequence of impaired CSPα chaperone activity. Clearly, it is important that further studies of post-mortem tissue from a variety of neurodegenerative diseases are performed to establish if altered CSP activity and/or SNARE complex assembly represent common underlying features of neurodegeneration.

## 6 Therapeutic implications

CSP is expressed in all synapses and prevents neurodegeneration in worms, flies, mice and humans. It therefore appears to be a generally neuroprotective chaperone protein. Drugs that could either increase CSP activity or bypass the requirement for CSP thus represent potential therapies – not only for ANCL, but also more generally for neurodegenerative diseases. Indeed, recent work has begun to identify compounds that can compensate for the lack of functional CSP in animal models. Treatment of CSPα knock-out mice with proteasome inhibitors was found to result in increased life-span and a delay in neurodegeneration [92]. This striking result is at first glance surprising, as one widely proposed therapeutic strategy for protein misfolding disorders that lead to neurodegeneration is to increase the activity of the ubiquitin-proteasome system that degrades the misfolded protein(s). It might be expected, therefore, that proteasome inhibition would exacerbate neurodegeneration in CSPα knock-out mice. The fact that the reverse is true was attributed to an observed increase in SNAP-25 levels and in assembled SNARE complexes through prevention of SNAP-25 degradation by the proteasome [92]. Furthermore, Sharma et al. showed that the reduced levels of assembled SNARE complexes that ultimately cause neurodegeneration in CSPα knock-out mice are also evident in post-mortem brain samples from Alzheimer's and Parkinson's disease patients [92]. Proteasome inhibitors may therefore have therapeutic applications for these common neurodegenerative diseases as well as for ANCL.

**Fig. 3:**
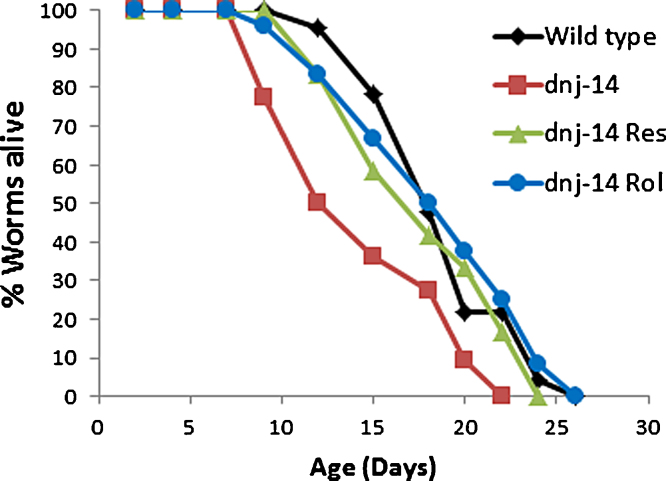
Resveratrol and rolipram rescue the short lifespan of *C. elegans dnj-14* mutants. Lifespan assays were performed on *dnj-14(tm3223)* strains, which contain a large deletion in the worm orthologue of CSP, in the presence and absence of drugs; and also on the corresponding wild type strain (Bristol N2).

A recent chemical screen revealed that resveratrol can rescue the short lifespan, chemosensory impairment and neurodegeneration phenotypes of *C. elegans dnj-14* null mutants [24]. Resveratrol is a plant-derived polyphenolic compound found in various foodstuffs, notably red wine, which has well-documented cardioprotective, anti-inflammatory, anti-tumour and neuroprotective properties [93]. Its mechanism of action is the subject of considerable debate, although most attention has focused on resveratrol's ability to activate the Sirtuin class of NAD+-dependent histone deacetylases, notably SIRT1. However, rescue of the *dnj-14* phenotypes by resveratrol was unaffected by deletion of the worm SIRT1 orthologue, *sir-2.1*, suggesting a Sirtuin-independent mechanism of action. It has been suggested that resveratrol is a competitive inhibitor of cAMP phosphodiesterases [94]. This is consistent with the observation that rolipram (a structurally unrelated cAMP phosphodiesterase inhibitor with neuroprotective properties [95,96]) similarly rescued *dnj-14* phenotypes (Fig. 3) [24]. Furthermore, forskolin, which increases cAMP levels via activation of adenylate cyclase rather than phosphodiesterase inhibition, ameliorates the neuromuscular transmission defects in CSPα knock-out mice [48]. It is tempting to speculate that increased cAMP signalling activates alternative CSP-independent pathways that facilitate SNARE complex formation, thereby compensating for the lack of CSP in these animal models. Indeed, phosphorylation of SNAP-25 and other synaptic exocytosis proteins by cAMP-dependent protein kinase is known to increase SNARE-dependent neurotransmission [97,98]. Alternatively, it may be that cAMP acts via longer term changes in gene expression mediated by neuroprotective transcription factors such as cAMP response element binding protein (CREB) [99]. Given the recent observations of reduced levels of CSP [90] and of SNARE complexes [92] in Alzheimer's brain samples, this may be relevant to the reported therapeutic effects of rolipram [95] and resveratrol [100] in rodent Alzheimer's models.

## 7 Conclusions

Despite being discovered over 20 years ago, interest in CSP has increased greatly in recent years as a result of compelling evidence that it provides a universal neuroprotective role at synapses from worms to humans. The major mechanism by which CSP prevents neurodegeneration appears to be maintaining the conformation of SNAP-25 and thereby facilitating correct SNARE complex formation. However, we currently lack a molecular level description of how CSP and Hsc70 interact with SNAP-25, how this alters SNAP-25's conformation to facilitate SNARE complex formation, and how this interaction (which presumably can only occur in close proximity to the very small number of docked synaptic vesicles at the active zone) manages to prevent global degradation of SNAP-25 that mainly localises away from this region. In addition it seems likely that other client proteins, including dynamin, are also involved, so future genetic and proteomic studies may illuminate such SNAP-25-independent functions of CSP. To date, mutations in the *DNAJC5* gene encoding CSPα have only been associated with ANCL, but given the alterations in CSP and SNARE complex levels in Alzheimer's disease, future human genetic studies may well reveal additional mutations/polymorphisms associated with other more common neurodegenerative disorders. Finally, the few studies reported so far demonstrate that it is possible to compensate for the lack of CSP with small molecules – further work in this area may enable translation of the basic research on CSP performed over the last 20 years into novel therapies for neurodegeneration.
